# Foraging Habitat Quality Constrains Effectiveness of Artificial Nest-Site Provisioning in Reversing Population Declines in a Colonial Cavity Nester

**DOI:** 10.1371/journal.pone.0058320

**Published:** 2013-03-06

**Authors:** Inês Catry, Aldina M. A. Franco, Pedro Rocha, Rita Alcazar, Susana Reis, Ana Cordeiro, Rita Ventim, Joaquim Teodósio, Francisco Moreira

**Affiliations:** 1 Centro de Ecologia Aplicada Prof. Baeta Neves, Instituto Superior de Agronomia, Universidade Técnica de Lisboa, Tapada da Ajuda, Lisboa, Portugal; 2 School of Environmental Sciences, University of East Anglia, Norwich, United Kingdom; 3 ICNF/PNVG - Instituto de Conservação da Natureza e Florestas/Parque Natural do Vale do Guadiana, Mértola, Portugal; 4 League for the Protection of Nature (LPN), Environmental Education Centre of Vale Gonçalinho, Castro Verde, Portugal; 5 Praça Capitães de Abril, Cova da Piedade, Portugal; 6 Estrada Mineiro, 16, A-dos-Bispos, Vila Franca de Xira, Portugal; 7 Calle Francisco de Icaza, Madrid, Spain; 8 Portuguese Society for the Study of Birds (SPEA), Nordeste, Açores, Portugal; Phillip Island Nature Parks, Australia

## Abstract

Among birds, breeding numbers are mainly limited by two resources of major importance: food supply and nest-site availability. Here, we investigated how differences in land-use and nest-site availability affected the foraging behaviour, breeding success and population trends of the colonial cavity-dependent lesser kestrel *Falco naumanni* inhabiting two protected areas. Both areas were provided with artificial nests to increase nest-site availability. The first area is a pseudo-steppe characterized by traditional extensive cereal cultivation, whereas the second area is a previous agricultural zone now abandoned or replaced by forested areas. In both areas, lesser kestrels selected extensive agricultural habitats, such as fallows and cereal fields, and avoided scrubland and forests. In the second area, tracked birds from one colony travelled significantly farther distances (6.2 km ±1.7 vs. 1.8 km ±0.4 and 1.9 km ±0.6) and had significant larger foraging-ranges (144 km^2^ vs. 18.8 and 14.8 km^2^) when compared to the birds of two colonies in the extensive agricultural area. Longer foraging trips were reflected in lower chick feeding rates, lower fledging success and reduced chick fitness. Availability and occupation of artificial nests was high in both areas but population followed opposite trends, with a positive increment recorded exclusively in the first area with a large proportion of agricultural areas. Progressive habitat loss around the studied colony in the second area (suitable habitat decreased from 32% in 1990 to only 7% in 2002) is likely the main driver of the recorded population decline and suggests that the effectiveness of bird species conservation based on nest-site provisioning is highly constrained by habitat quality in the surrounding areas. Therefore, the conservation of cavity-dependent species may be enhanced firstly by finding the best areas of remaining habitat and secondly by increasing the carrying capacity of high-quality habitat areas through safe nest-site provisioning.

## Introduction

Maintaining or increasing the population numbers of endangered species requires the identification of the limiting factors of population sizes, without which the management of any species population is likely to be unpredictable [1]. Within their habitats, most bird populations are naturally limited by the availability of food, safe nest-sites, predation, competition and diseases [2]. Nonetheless, the carrying capacity of any environment for breeding populations is usually set by two resources of major importance - nest-sites and food - and whichever is in shortest supply can limit the number of breeding pairs [2]. Inadequate quantity or quality of food may prevent or significantly depress breeding by impacting parameters such as laying date, productivity or juvenile survival (e.g. [3]–[6]) and has been pointed out as the main driver of population declines in several bird species (e.g. [7]–[9]). On the other hand, nest-site shortage can prevent individuals from breeding, influence the number of breeders and non-breeders and, therefore, the total population size [10]–[12]. Secondary cavity bird nesters are unable to excavate their own cavities and are strongly limited by the availability of natural nest-sites nearby suitable foraging areas. As a result, nest-box programmes have been often recommended as a conservation tool for several rare and endangered cavity-nesting species [13]. Nonetheless, the interaction between food and nest-site availability seems to play a decisive role during the breeding season: the distance that birds travel between nest-sites and foraging areas seems to depend largely on food availability and constitutes an important component of time-energy budgets [14]–[17]. Foraging ranges are thus determined largely by the number of feeding places used and by the distance between them. The energetic costs of nesting a long distance from foraging grounds tend to be inefficient and may have ecological consequences resulting in poor breeding success [6], [14].

In the European Union, member states are bound by the Birds Directive (79/409/EEC and 2009/147/EC) to improve the conservation status of bird species by protecting or enhancing their habitats through the establishment of a coherent network of Special Protection Areas (SPAs). Despite the measurable conservation benefits of one such instrument [18], conservation investment is highly constrained and today’s most important challenge is to prioritize activities to allocate scarce funding and resources effectively [19]–[21], avoiding, for example, conflicting and contradictory measures promoted by different financial instruments [22]. To achieve this, conservation planning of target species should firstly assess species limiting factors, such as habitat quality and nest-site availability and its impacts on the breeding success and population demography.

The lesser kestrel *Falco naumanni* is a colonial falcon closely associated with open agricultural landscapes. Breeding pairs can be found nesting in colonies of up to 200 pairs [23], occupying old buildings (such as castles or churches) in small villages or towns and isolated abandoned farmhouses in the countryside. Lesser kestrels forage predominantly on insects in steppe-like habitat, natural and managed grasslands and low-intensive agricultural areas [24]. The western European population has sharply declined by about 95% between 1970 and 1990 [25].

The documented decline has been mostly associated with habitat loss, through agricultural transformation, predation and human disturbance and loss of nest-sites, often related with restoration, demolition or collapse of old buildings [26]. Lesser kestrels are obligatory cavity nesters and loss of nest-sites has lead to colony reduction or desertion [27]–[29]. Moreover, shortage of suitable nest-sites has been suggested to limit population growth in several areas [30]–[32] and nest-site provisioning the most effective measure to ensure population persistence in other areas [31], [33].

During recent decades extensive farmland landscapes have changed rapidly as a result of the Common Agricultural Policy (CAP). In the best soil areas, agriculture has been intensified drastically and seems to be responsible for reducing food availability for kestrels, as preferred hunting habitats are being replaced by others where prey are scarcer and/or kestrels hunt them less efficiently [17], [32], [34]. Larger foraging ranges, high chick mortality due to starvation and colony desertion have been suggested to be likely consequences of these land-use changes [28], [35]. Paradoxically, intensification has also lead to agricultural abandonment in less productive areas often with afforestation of former agricultural fields. The consequences of these changes for lesser kestrels have not yet been fully assessed.

Recent evidence indicates a stable or slightly positive population trend overall during the last three generations. Consequently it has been downlisted from Vulnerable and now qualifies as Least Concern [25]. It is listed in Annex I of the European Birds Directive.

In this study we examine how differences in foraging habitat quality and nest-site availability in three colonies in two Special Protection Areas (SPA) in southern Portugal affected the breeding success and population trends of two lesser kestrel populations. Both areas have benefitted from management interventions to increase nest-site availability [17] but the variations in the quality of the surrounding foraging habitats across time is contrasting: one area (two rural colonies) is dominated by unchanged pseudo-steppe habitat characterized by traditional agricultural practices of extensive cereal cultivation whereas in the other (with one urban colony) traditional agricultural areas have been abandoned or replaced by forested areas. We used data from a long-time monitoring program of the Portuguese lesser kestrel population and telemetry data to investigate differences in (1) patterns of habitat availability and use, (2) foraging behaviour, (3) nest-site occupation and (4) breeding performance between the two areas. If agricultural transformation in one area affects habitat quality around the colony, reducing prey accessibility and preferred hunting habitats, it is expect that kestrels would have to travel longer distances and have larger foraging home-ranges. We predict that habitat quality between the two sites would result in differences in provisioning rates for nestlings, chick body condition and breeding success. Moreover, if recent changes in agricultural practices have contributed to the decline of the species, the habitats most favourable for the species should have suffered the largest reduction. To test this hypothesis we examine historical changes in land-use and lesser kestrel population trends in the two areas. Finally, we estimate predation rate in the three colonies to investigate whether kestrels trade off lower predation pressure and access to foraging grounds when living in safer urban environments as suggested by Tella *et al*. [36]. Based on our findings we discuss management implications for increasing the effective conservation of lesser kestrels and other bird cavity nesters.

## Methods

### Ethics Statement

The deployment of transmitters (see details below) did not take more than 15 minutes and on no occasion did it interfere with reproduction or have visible deleterious effects on study animals. All work (telemetry and ringing) was approved by the relevant authorities (Instituto da Conservação da Natureza e da Biodiversidade; research permits 85/2000, 2 & 96/2001, 2 & 102/2002, 2 & 46 & 100/2003, 48 & 105/2004, 48 & 107/2005, 50 & 110/2006, 55 & 120/2007). To access colonies all land owners were asked for permission; for monitoring lesser kestrels’ nests no other specific permissions are required.

### Study Area

The study was conducted in the Castro Verde and Vale do Guadiana Special Protection Areas (SPAs), two contiguous areas with 85 000 and 76 000 hectares in southern Portugal. In the Castro Verde plains the landscape is dominated by extensive cereal cultivation characterized by a mixture of fallows and cereals, leguminous crops and ploughed fields. Since 1995, farmers in this area can apply voluntarily to an agri-environmental scheme that aims to maintain favourable feeding habitat for a range of steppe bird species, including the lesser kestrel. Here, lesser kestrel colonies are located either in old adobe-built abandoned farmhouses (nests are located in cavities in walls or in the roof, under the tiles) or artificial nesting structures built with LIFE-nature funds [17]. Belver and Pardieiro colonies, 30 km apart from each other and located in ruins of abandoned farmhouses, hold the largest colonies of this area and were selected to this study. The Vale do Guadiana SPA includes the only urban colony in Portugal (in the Mértola village). Lesser kestrel pairs are found in natural cavities of the medieval walls that surround the village and in artificial nests specifically provided for the species. In this area, most of the traditional extensive agricultural areas are being abandoned (leading to scrub encroachment) or replaced by pine plantations. Other habitats include orchards, holm and cork oak open woods and eucalyptus.

### Land-use Changes

To assess the degree of land use changes around the studied colonies we used a detailed land cover and land use map of Portugal for the year of 1990 (COS’90), produced in vector format at the scale 1∶25 000 [37] and information collected in the field for the year of 2002. All information was integrated in a Geographic Information System using ARCVIEW 3.2 [38] and land use changes between 1990 and 2002 were calculated for a radius of 3 km around each colony. This radius was chosen because during the nestling period most foraging trips take place in this buffer around colonies [32], [39]. The land use classes defined in COS’90, were pooled into 12 categories in order to simplify comparisons (Table 1). Moreover, we re-classified each class according to its suitability as foraging grounds for lesser kestrels based in the literature [32], [34], [39], [40]–[41]. Suitable habitat included extensive agricultural areas such as cereals, stubbles, fallows, ploughed fields and small areas of leguminous crops. Marginal habitats included open scrubland and montado areas and recent forestations (wooded areas are know to be rejected by lesser kestrels; e.g. [34]) and unsuitable habitats included all other habitats such as forest, orchards and horticultural areas, water and human infrastructures (Table 1).

**Table 1:** Habitat classes within a 3 km radius around the studied lesser kestrel colonies.

Habitat	Description	Suitability for lesser kestrels
extensive agricultural areas	extensive cultivation of cereal characterised by a mixture of grazed fallow areas androtations of cereals, ploughed fields, leguminous crops and stubbles	suitable
open scrubland	extensive agricultural areas with 30–50% of scrub cover	
open montado	open holm and cork oak woods (<30% cover)	marginal
recent forestations	holm oak and pine plantations with less than five years	
scrubland	scrub encroachment areas with >30% cover	
irrigated areas	irrigated crops such as corn, sunflower or beet	
montado	holm and cork oak woods (>30% cover)	
forest	pine and eucalyptus forest	unsuitable
orchards	orchards and olive	
horticultural areas	small orchards and gardens	
urban areas	urban areas and human infrastructures	
water	rivers, ponds, etc	

Habitats were also classified regarding its suitability for lesser kestrels (see methods).

### Nest-site Provisioning, Occupation Rates and Population Trends

Artificial nest-sites were provided from 1985, 1996 and 2004 in the Mértola, Belver and Pardieiro colonies respectively, and included new cavities opened in existing buildings, wooden nest-boxes, clay pots and breeding towers (concrete-built structures with many available cavities). From 1995 to 2007, colony size (number of breeding pairs that layed at least one egg) and occupation rate was assessed in each breeding season from several visits in which all potential nest-sites were checked for occupation.

### Foraging Habitat and Behaviour

A total of 33, 30 and 25 adult lesser kestrels were radio-tracked in Belver (in 2000), Pardieiro (in 2004) and Mértola (in 2000) colonies, during the breeding season. Transmitters from Biotrack, weighing 4.2 g, were used in 2000 while in 2004 we used Holohil transmitters of 3.8 g. All devices represented less than 3% of the weight of an adult and were tied dorsally to the base of two central tail feathers [42]. Similarly to other studies using the same type of radios on this species [43], we found no negative effects on either survival or reproductive success from the use of the transmitters [38]. In the Castro Verde colonies (Belver and Pardieiro), kestrels were tracked by triangulation [42], [44] from three fixed telemetry stations built in strategic high points around each colony. With these fixed stations a 4.5 and 5 km area around the Belver and Pardieiro colonies, respectively, was completely covered. At least two of the stations were used in each session and walkie-talkies were used to ensure simultaneous locations of the transmitters’ positions. Details on tracking methodology followed are described in Franco et al. [39]. Contrarily to the flat areas around the Castro Verde colonies, the hilly landscape around Mértola did not allow for the use of fixed stations as a large part of the area would not be covered using this technique. Thus, in the Mértola colony tracking was performed by one observer situated at the colony that monitored the nests and communicated by radio every movement of tracked birds to another team that followed the birds in the terrain [32], [34]. Every time a bird was observed hunting, we recorded location and habitat use in 1/25 000 maps. Tracked birds were followed with mobile Yagi antennas with three elements and using a TRX 10S Wildlife Materials receiver. Tracking was carried out along the breeding season in the three colonies. To ensure independence of data, we separated each location of the same bird by an interval of at least 30 minutes in the Castro Verde colonies and 45 minutes in Mértola.

#### Habitat selection

Land use around lesser kestrel colonies during the telemetry study years was characterized from aerial photographs taken in 1995 (Belver and Pardieiro) and 1999 (Mértola) and confirmed by field data. Habitat availability was estimated within a 4 and 5 km radius around the Belver and Pardieiro colonies, respectively (where more than 90% of the telemetry locations were recorded) and within a 6 km radius around the Mértola colony (cartography was only available for this area, containing 80% of the telemetry locations). Habitat types present within this radius were classified in 6 classes: extensive agricultural areas (including cereals, fallows, ploughed fields, leguminous crops and stubbles), recent forestations (with less than 5 years), forest (pine and eucalyptus woods), scrubland, montado (holm and cork oak woods) and other (e.g. orchards and horticultural areas, urban areas, water courses). The few locations occurring at the intersection of habitats were discarded (see [39]). Habitat selection in each colony was analysed using the Savage selectivity index [45] 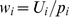, where 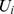 is the proportion of observations recorded in a given habitat and 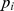 is the proportion of that habitat against total available habitat. This index varies from 0 (maximum negative selection) to 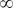 (maximum positive selection), 1 indicating no selection. The statistical significance of this index was tested by comparing the statistics 
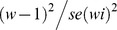
with the corresponding critical value of a chi-squared distribution with one degree of freedom [46]. The standard error of the index 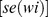 was calculated by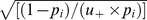, 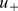 being the total number of foraging observations sampled. Statistical significance was obtained after applying the Bonferroni correction for the number of statistical tests.

#### Home ranges and foraging distances

Home-range areas were defined, using RANGES V [47] and CALHOME [48] according to the Minimum Convex Polygon method and including 90% of the locations [49]. This method was chosen because it is the only method that is strictly comparable between studies [50]. For each foraging observation, the distance between the colony and the location of the foraging bird was estimated. Comparisons between home-range size and foraging distances among the three colonies were assessed using Kruskal-Wallis followed by post hoc Tukey tests.

### Breeding Parameters

#### Chick feeding rates and prey size

We recorded chick feeding rates (number of food items brought to the nest per hour and adult) in each colony in 2000 and 2001 (in the Pardieiro colony observations were performed only in 2001). Observation periods were spread throughout the day with a minimum of 6 hours per nest. Comparisons of chick provisioning between colonies was done by pooling the two years together and using Kruskal-Wallis followed by post hoc tests. To assess differences in prey size delivered to the chicks we classified each item, whenever possible, as small, medium or large [17]. Data regarding prey size were only collected in 2000 at Belver and Mértola colonies.

#### Predation rate, fledging success and chick body condition

Predation was defined as the ratio of predated clutches or broods by the total number of clutches for the period 2003–2006. To assess breeding success we estimated fledging success - number of fledglings per nest where at least one chick fledged - in 2000 (only Belver and Mértola), 2003, 2004 and 2005. During these years, all potential nest-sites were monitored on a weekly basis to ensure an effective calculation of the breeding parameters. To compare predation rate and fledging success among the three colonies we pooled the data for the entire periods. Mean chick body condition was calculated from the residuals of a locally weighted regression (LOESS) between 8th primary feather length and body mass. It has been argued that such residuals provide the cleanest way to separate the effects of condition from the effects of body size (e.g. [51]). We considered biometrics of fledglings at age of ringing and found no differences in wing length between colonies (min = 100 and max = 195 mm;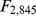 = 0.69, *p* = 0.5). Comparisons of chick body condition among colonies were assessed using a one-way ANOVA followed by post hoc Tukey tests using data collected in 1995, 1996, 1998, 2000 and 2003.

## Results

### Land-use Changes

The percentage of suitable foraging habitats (extensive farmed areas including fallows, cereal, stubbles and ploughed fields) around the Castro Verde colonies - Belver and Pardieiro - exceed 90% in 1990 and remained mainly unchanged until 2002 (Table 2). In contrast, the area of suitable foraging habitat surrounding the Mértola colony decreased from 32 to only 7% in the same period (Table 2). The loss of foraging habitat quality was a consequence of the replacement of extensively farmed areas by pine plantations scrubland areas due to land abandonment.

**Table 2:** Land-use changes within a 3 km radius around three lesser kestrel colonies between 1990 and 2002.

	Foraging habitat (%)					
	Suitable		Marginal		Unsuitable	
	1990	2002	1990	2002	1990	2002
Belver	0.94	0.89	0.02	0.08	0.04	0.04
Pardieiro	0.91	0.92	0	0.01	0.09	0.07
Mértola	0.32	0.07	0.13	0.38	0.54	0.55

Habitats were classified as suitable, marginal and unsuitable regarding lesser kestrels’ foraging preferences (see methods).

### Nest-site Provisioning and Population Trends

**Figure 1:**
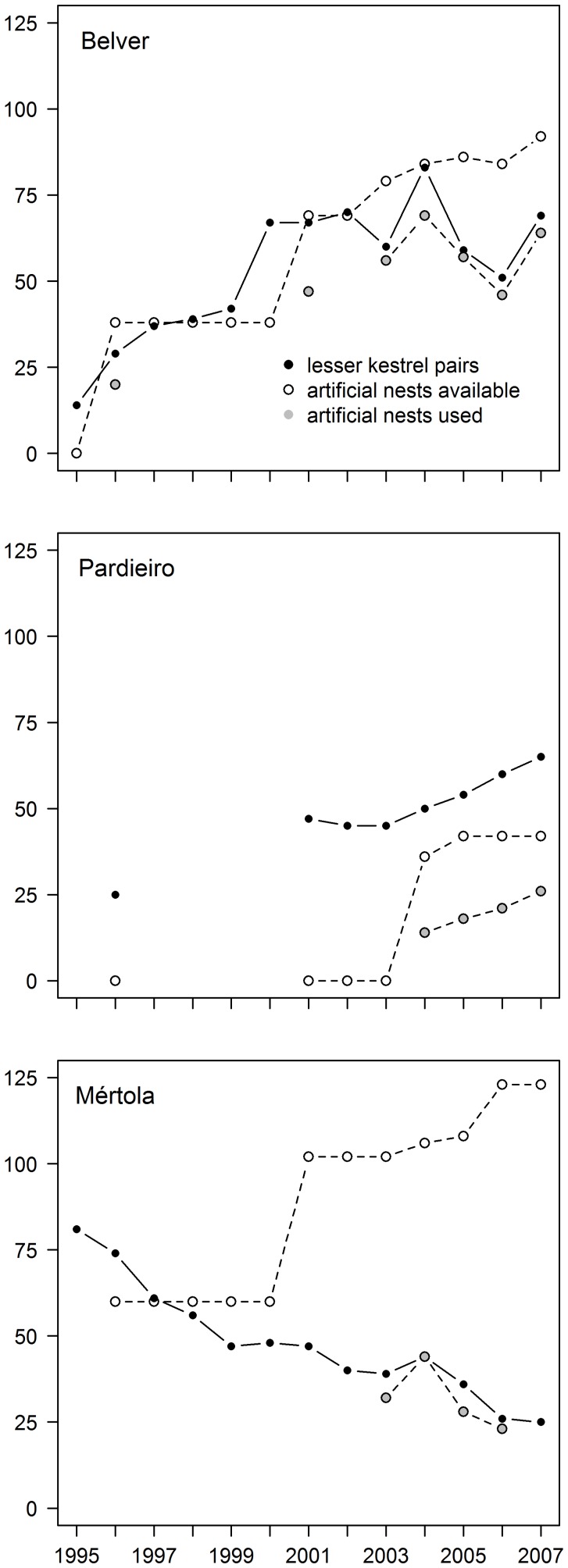
Evolution of lesser kestrel breeding pairs in Belver, Pardieiro (Castro Verde SPA) and Mértola (Vale do Guadiana SPA) colonies. Minimum values of estimates for colony size (black cicles) and number of artificial nests available (white circles) and used (grey circles) in each year are shown.

In 1995, the Belver colony held only 14 pairs of lesser kestrels breeding under the roof tiles of abandoned buildings. Since 1996, the occupation of provisioning of artificial nest-sites (mainly cavities opened in the adobe walls) allowed the rapid and significant increase of colony size (Fig. 1). Between 1996 and 2007, 92 nest-sites were provided and the proportion of breeding pairs using artificial nests varied between 69 and 93%; the population increased up to 83 breeding pairs (Fig. 1). The Pardieiro colony was relatively stable between 2001 and 2004 showing an increase in the number of breeding pairs from 2004, when ca. 40 new cavities were provided (Fig. 1). In 2007, 40% of the population (26 out of 42 pairs) used the artificial nests provided. At Mértola and despite the provisioning of more than 120 artificial nests between 1995 and 2007, colony size decreased from 81 to 25 pairs in the same period (Fig. 1; most of pairs used artificial nests). Belver is currently the largest lesser kestrel colony in Portugal (81 pairs in 2012) while Pardieiro is the second largest (71 pairs in 2011). Mértola was the largest colony until 1999 but currently more than 10 colonies in the Castro Verde SPA exceed the 25 pairs recorded in this colony in 2007.

### Foraging Habitat Selection

**Figure 2:**
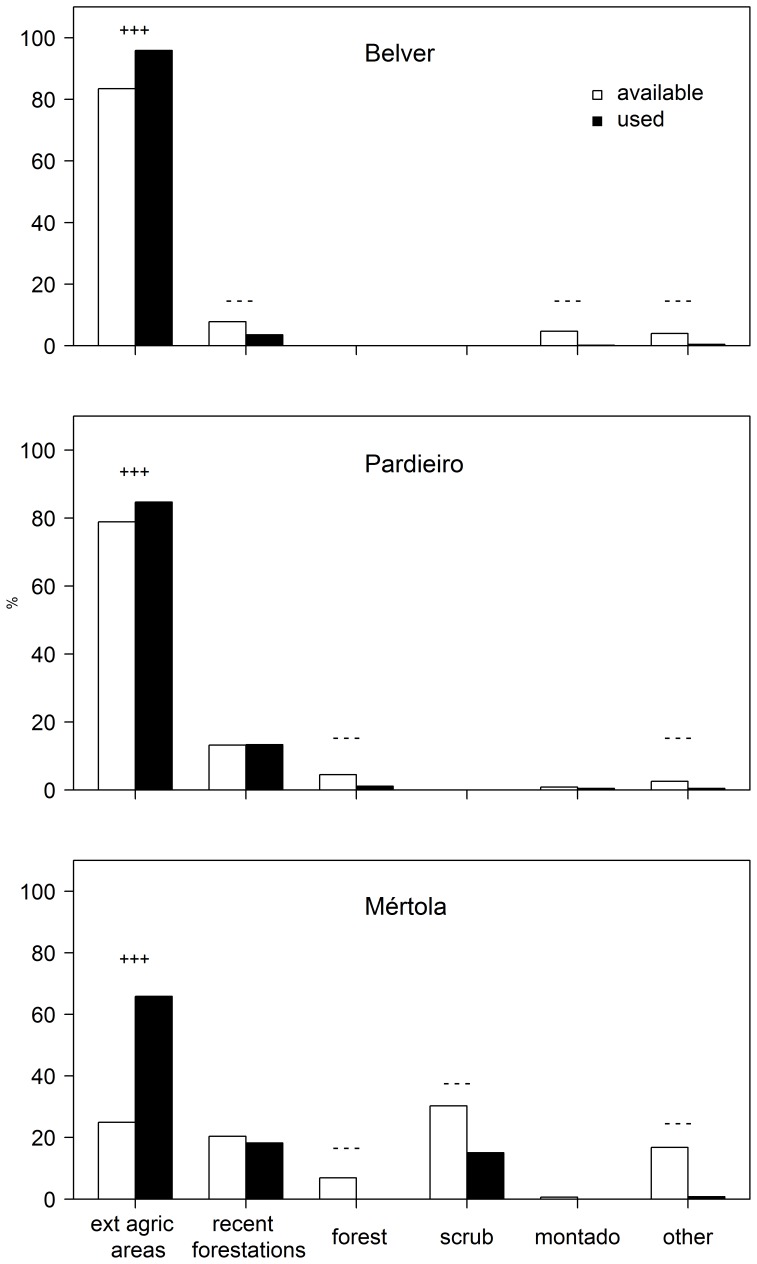
Foraging habitat selection of lesser kestrels in Belver (n = 684) and Pardieiro (n = 457) and Mértola (n = 126) colonies. Percentage of available (white) and used (black) habitats by foraging lesser kestrels are presented.

A total of 1267 foraging observations were recorded in the three colonies (n = 684, 457 and 126 for Belver, Pardieiro and Mértola, respectively). Lesser kestrels positively selected extensive agricultural areas in all colonies (Belver: 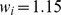 *p*<0.001; Pardieiro: 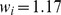 *p*<0.001 and Mértola: 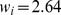 *p*<0.001, Fig. 2 and Table 3). Fallows and cereal fields, especially during harvesting were the most used habitats. Recent forestations (<5 years) were used less that expected in Belver (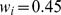, *p*<0.01) and in proportion to its availability in Pardieiro and Mértola colonies (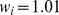 and 0.89, p>0.05, respectively) while the rest of the habitats were mainly avoided (Fig. 2 and Table 3).

**Table 3:** Values of Savage selectivity index (ω^i^), standard error (SE) and significance level (*P*) for each habitat used by lesser kestrels around the three studied colonies.

	ωi	SE	*P*
**Belver**			
extensive agricultural areas	1.15	0.02	<0.001
forestations (<5 years)	0.45	0.13	<0.01
montado	0.03	0.17	<0.001
others	0.11	0.19	<0.01
**Pardieiro**			
extensive agricultural areas	1.17	0.02	<0.001
forestations (<5 years)	1.01	0.12	n.s.
forest	0.24	0.22	<0.01
montado	0.51	0.50	n.s.
others	0.17	0.29	<0.05
**Mértola**			
extensive agricultural areas	2.64	0.15	<0.001
forestations (<5 years)	0.89	0.18	n.s.
forest	0.00	0.33	<0.05
scrubland	0.50	0.14	<0.01
montado	0.00	1.09	n.s.
others	0.05	0.20	<0.001

### Home-ranges and Foraging Distances

**Figure 3:**
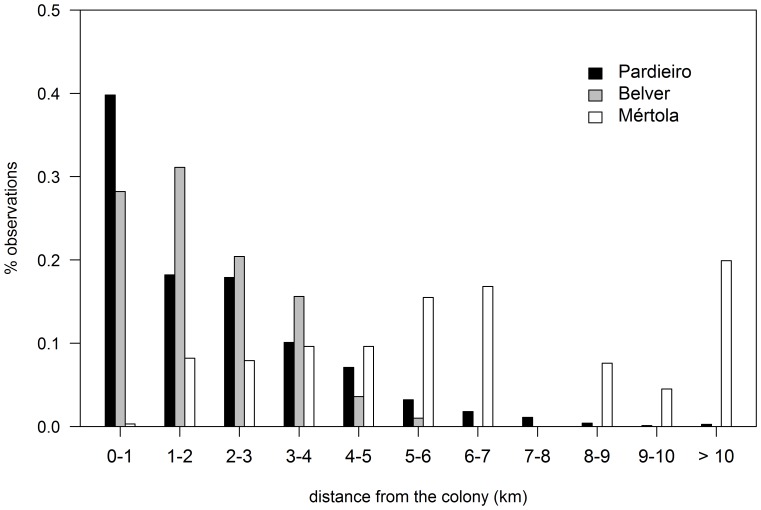
Distribution of foraging locations of lesser kestrels in relation to the distance from the colony at Belver, Pardieiro and Mértola. Foraging trips were significantly longer in Mértola (mean distance = 6.24 km ±1.73, n = 291) than in the Castro Verde colonies (Belver = 1.84 km ±0.37, n = 588 and Pardieiro = 1.92 km ±0.61, n = 814).

Mean home ranges of individuals tracked in the three colonies varied considerably: while in Belver and Pardieiro the mean home ranges obtained using the MCP method were 18.8 and 14.8 km^2^ respectively, the mean home-range estimated in Mértola was 144 km^2^ (Table 4). Mean home-ranges were significantly different among colonies for males, females and all individuals, showing significant differences between Mértola and Castro Verde (Pardieiro e Belver; Table 4). Similarly, mean distance travelled by kestrels to foraging grounds was significantly different among colonies (Kruskal-Wallis 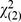 = 38.4, p<0.001 followed by post hoc Tukey tests), showing significant differences between Mértola (mean distance = 6.24 km ±1.73, n = 291) and the Castro Verde colonies (Belver = 1.84 km ±0.37, n = 588 and Pardieiro = 1.92 km ±0.61, n = 814). During the breeding season, 82% of foraging locations in Belver and 78% in Pardieiro were within a radius of 3 km around the colony whereas in Mértola only 32% of the foraging locations were recorded in the same radius (Fig. 3). At Mértola, tracked individuals were located in foraging patches at a maximum distance of 15.4 km from the colony.

**Table 4:** Comparative home-range size of lesser kestrels in the three studied colonies derived using the Minimum Convex Polygon (MPC 90%) method.

	Home-range (km^2^)				
	Belver	Pardieiro	Mértola	Test	*P*
**MCP 90%**					
Females	14.9±2.7^a^	16.6±8.1^a^	123.0±6.3^b^	χ^2^_(2)_ = 12.3	<0.01
Males	23.4±7.0^a^	13.5±5.1^a^	165.1±31.9^b^	χ^2^_(2)_ = 9.2	<0.05
All	18.8±6.6^a^	14.8±6.3^a^	144.0±30.1^b^	χ^2^_(2)_ = 19.8	<0.001
**min-max**					
Females	9.9–17.9	6.1–28.8	160.6–169.5		
Males	12.9–32.1	4.3–22.7	81.4–146.2		
All	9.9–32.1	4.25–28.8	81.4–169.5		
**number of locations**					
Females	55±13.1 (7)	30±6.1 (8)	27±2.6 (4)		
Males	77±15.8 (6)	39±13.9 (13)	28±3.5 (2)		
All	66±18.0 (13)	35±11.7 (18)	27±2.7 (6)		

Mean (± SD), maximum and minimum home-ranges, number of locations and sample sizes (in brackets) are shown. Columns sharing different letters (superscripts) are significantly different.

### Breeding Parameters

Chick feeding rates were significantly different among colonies (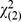 = 19.7, p<0.001, followed by post hoc Tukey tests) being significantly lower in Mértola than in the Castro Verde colonies (Table 5). Prey size delivered to chicks was significantly different in Belver and Mértola colonies with adults of Mértola delivering a higher proportion of small prey when compared to Belver (Table 5).

**Table 5:** Comparative breeding parameters of lesser kestrels in the three studied colonies.

	Castro Verde SPA		V. Guadiana SPA		
	Pardieiro	Belver	Mértola	Test	*P*
**Chick feeding rates**	2.30±1.16^a^ (n = 30)	1.71±0.75^a^ (n = 61)	1.17±0.34^b^ (n = 21)	χ^2^_(2)_ = 19.7	<0.001
**Prey size (proportion)**					
small	–	0.07	0.18		
medium	–	0.35	0.33	W = 16105	<0.05
large	–	0.57	0.49		
n	–	374	76		
**Chick body condition**	7.21±1.05^a^ (n = 261)	3.19±0.86^a^ (n = 392)	−16.09±1.47^a^ (n = 195)	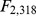 = 107	<0.001
**Fledging success**	3.15±0.2^a^ (n = 102)	2.56±0.23^b^ (n = 201)	2.02±0.68^c^ (n = 79)	χ^2^_(2)_ = 31.9	<0.001
**Predation rate**	0.04±0.4 (n = 213)	0.19±0.11 (n = 201)	0.09±0.03 (n = 86)	χ^2^_(2)_ = 21.6	<0.001

Chick feeding rates were calculated as the number of prey delivered per adult and hour (number of nests sampled in brackets). Chick body condition is shown as the mean residuals of a locally weighted regression (LOESS) between 8th primary feather length and body mass, fledging success as the mean number of fledglings per successful pairs and predation rate as the ratio of predated clutches or broods. Mean values (± SD) and sample sizes are shown. Columns sharing different letters (superscripts) are significantly different (p<0.01).

**Figure 4:**
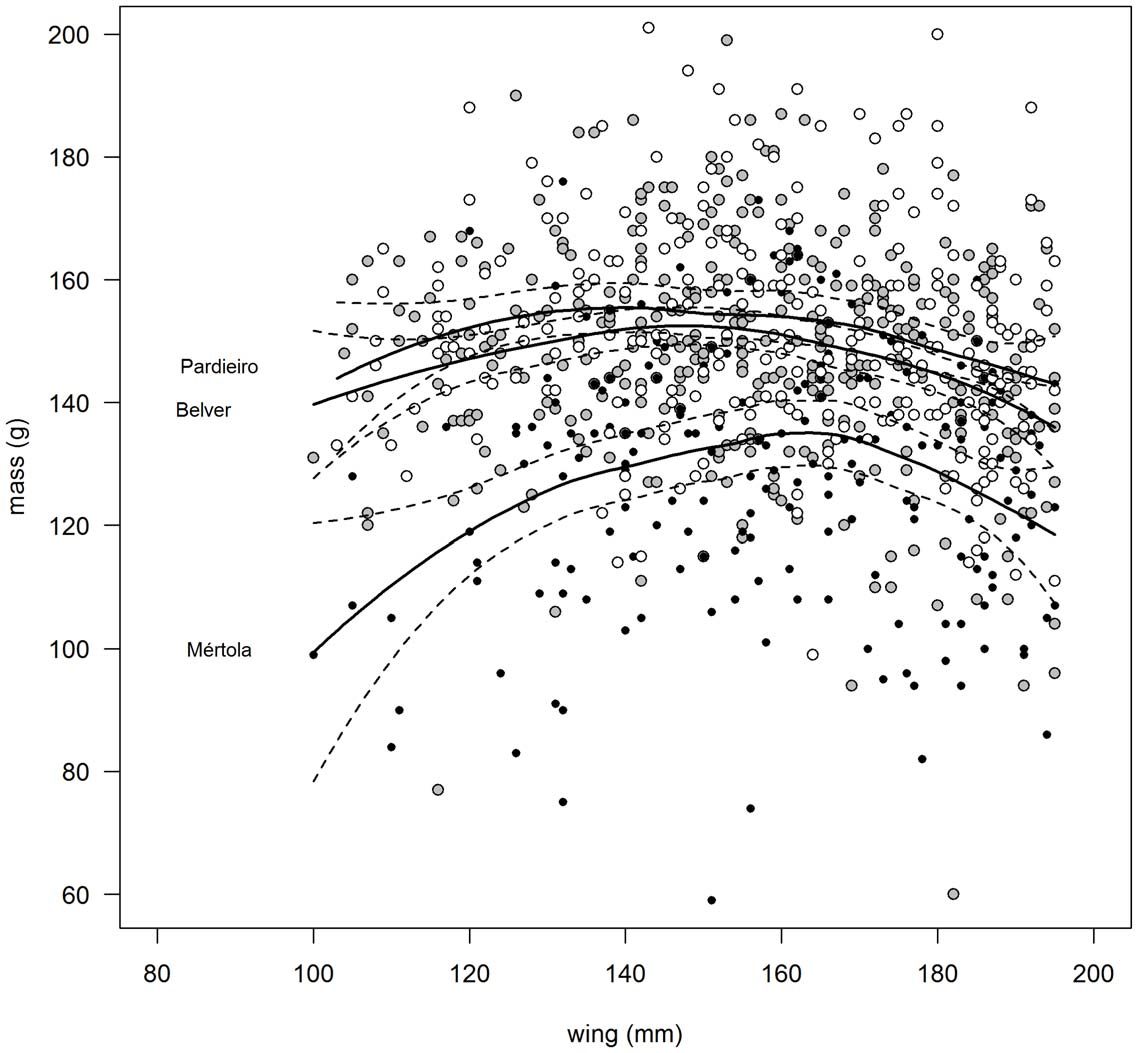
Chick body mass (g) and wing length (mm) of lesser kestrels (n = 848) in the three studied colonies. The trend lines were estimated using LOESS and dashed lines represent 95% confidence intervals.

Predation rate varied between colonies showing intermediate values in the urban colony of Mértola (Table 5). Mean number of fledglings per pair was significantly different among all colonies, with Mértola holding the lower value, followed by Belver and Pardieiro (Table 5). Chick body condition was significantly lower in Mértola when compared to the Castro Verde colonies (Fig. 4, Table 5).

## Discussion

### Influence of Habitat Quality on Foraging and Breeding Performance of Lesser Kestrels

Our results showed that during the breeding season lesser kestrels preferentially forage in agricultural extensive areas, such as fallows and cereal fields, which is in accordance with others authors’ findings (e.g. [34], [52]–[53]). As the proportion of suitable feeding habitats around the Mértola colony decreased, the selection index for this habitat increased, confirming that birds progressively concentrate in the remaining suitable patches as feeding conditions deteriorate. At the same time, marginal habitats that were clearly avoided in the best feeding areas (Castro Verde colonies) were more intensively used in the area surrounding the Mértola colony. The low availability of preferred habitats around the Mértola colony constrained the foraging efficiency of lesser kestrels in several ways. First, foraging trips of individuals from Mértola were more than three times longer than those from the Castro Verde colonies, presumably reflecting insufficient food resources around Mértola and the need for searches in suitable foraging habitat patches farther away from the colony. In fact, lesser kestrels in Mértola had very large foraging ranges (∼100 km^2^), exceeding the ones previously reported for this species either in traditional agro-grazing systems [34] or partial intensively cultivated areas [32], [35]. Secondly, longer foraging trips were reflected in lower chick feeding rates. Thirdly, these longer trips were not compensated by the delivery of larger prey - on the contrary there was a higher frequency of smaller prey delivered to chicks in Mértola, probably reflecting the lower hunting efficacy in marginal habitats – or by a lower predation risk. Tella et al. [36] suggested that lesser kestrels of urban colonies (such as Mértola), where nests are usually located farther way from foraging grounds when compared to rural colonies, could benefit from reduced predation pressure provide by safer urban environments and thus kestrels could face a trade off between lower predation risk and distance to foraging sites. Our results do not fully support this idea as predation was higher in Mértola when compared with one of the rural colonies. Mértola is a small village surrounded by agricultural fields and easily accessible to most predators. Indeed, potential predators of lesser kestrels’ nests, such as rats, snakes and barn-owls, were observed in both rural and urban areas. Moreover, rural areas become safer places for lesser kestrels in the last decade as suggested by the observed decline in predation rate due to the provisioning of adequate artificial nests [31]. The establishment of this large colony is more likely related with the extinction of another colony (due to the restoration of an old castle where the kestrels nested, [27]) and with the low availability of breeding sites in the surrounding rural areas prior to nest-site provisioning [31]. Overall, lower intake rates and smaller prey resulted in poorer chick body condition (a good indicator of juvenile survival probability; [54]) and lower fledging success at Mértola. Chick starvation and low breeding success have been previously pointed as possible consequences of agricultural intensification [32], [35]. Despite the fact that most observed declines on farmland bird populations are attributed to factors linked to the intensification of agriculture, population declines in arable landscapes have also been linked to land abandonment and afforestation [55]–[56]. Our results support this latter explanation, showing that land abandonment may have negative consequences on lesser kestrel breeding performance.

### Impacts of Nest-site Provisioning for Lesser Kestrel Demography

Nest-site limitation is particularly acute for cavity-dependent species which can be excluded or kept at low density levels where nest-sites are scarce [11]. The lack of suitable nest-sites was identified as the main limitation of the lesser kestrel Portuguese population [30] and the massive provision of artificial nest-sites the main responsible for its rapid recovery [31]. By 2007, 52% (n = 279 pairs) of the whole breeding population used artificial nests [31] and at our studied colonies this percentage was even higher (see results and Fig.1). The national census carried out in 2007 showed an increase in average colony size (as in Belver and Pardieiro, Fig. 1) and in the number of colonies of the Castro Verde SPA (this area held 50%, 60% and 80% of the Portuguese population in 1996, 2001 and 2007, respectively). Nonetheless, and despite the high number of available nest-sites, lesser kestrels at Mértola showed a negative population trend, declining from 81 to 25 breeding pairs in 13 years and with the occupation rate of artificial nests never exceeding 50% (Fig. 1). The importance of the Vale do Guadiana SPA for the national population decreased from 50% to 16% and 5% during the same period, reflecting the population decline in the Mértola colony.

### Limiting Factors of Population Size in Lesser Kestrels: Nest-site Availability or Habitat Quality?

In the absence of nest-site limitation, the detrimental effects of the progressive deterioration in habitat quality (suitable habitat decreased from 32% in 1990 to only 7% in 2002) are likely to be the main drivers for the observed population decline at Mértola colony. Land abandonment around Mértola resulted in the increase of the scrubland areas (by loss of grazing) and active afforestation. While these habitats can be used by lesser kestrels in the beginning of the vegetation succession or when trees are small (forestations with less than 5 years were used in proportion of its availability in two out of the three studied colonies), scrubland and wooded areas are known to be avoided by lesser kestrels [32], [52]. Therefore, the habitat classified as marginal will become unsuitable in a short to medium term period reducing even more the future foraging options of the breeding pairs. A contrasting situation was found in the Castro Verde SPA where the high availability of suitable foraging habitats within a 3 km radius around the colonies favours foraging lesser kestrels. The maintenance of good quality habitats inside this SPA is a consequence of the policies applied in the area that include the interdiction of afforestations and the existence of an agri-environment scheme (AES) to promote the traditional extensive cultivation of cereals in a rotational system. This AES guarantees a large area of fallow land, the reduced use of pesticides and herbicides and the control of grazing intensity. The habitat quality of our study colonies in the Castro Verde SPA has been pointed out as high enough to guarantee the persistence of this lesser kestrel population [17]. Although with high quality foraging areas, the steep increase in population size recorded in our colonies and in the whole SPA was only possible after the provisioning of artificial nests [31]. Thus, our results suggest that not only nest-site availability or habitat composition around the colonies but rather the interaction between these two factors are likely to play a major role in the limitation of breeding density and reproductive output of lesser kestrels.

### Management Implications for Lesser Kestrels and Other Cavity Nesters

The delivery of environmental benefits through the implementation of SPAs has been poorly evaluated but some studies suggest that the existent network does not cover adequately the most important areas for birds excluding, for example, crucial foraging habitats sites [57]–[59]. Despite the overlap between the distribution of the Portuguese lesser kestrel population and the national SPA network as well as the conservation measures implemented, opposite population trends have been recorded inside different protected areas (authors’ unpublished data). For many species, including the lesser kestrel, a shortage of nest-sites has been rectified by adding sites artificially (e.g. [11], [60]–[61]), often without assessing the quality of surrounding foraging habitats. As species limited by nest-site location cannot respond to environmental changes simply by changing nest location they may experience greater limitations under habitat loss. Therefore, from a conservation perspective, our results suggest that hole-dependent species management may be enhanced by (1) finding the best areas of remaining habitat and (2) increasing the carrying capacity of these target areas through, for example, nest-site provisioning. We should stress that artificial nests should be added in the proximity of high quality foraging grounds taking into account the foraging requirements of target species [17]. Moreover, nest-sites should be suitable for the target species guaranteeing the protection from predators, inclement weather and competitors [29], [31], [36]. As for the lesser kestrel, high quality foraging habitat may be compromised in the Castro Verde SPA by the decline in the financial support to farmers’ participation in agri-environment schemes (AES), a widely accepted management tool regarding the conservation of farmland landscapes [62]–[63]. The recorded funding reduction already account for a significant decline in the area affected, from 61% in 1999 to under 15% by 2007 [64] and may jeopardize the maintenance of the most important Portuguese region for the lesser kestrel. Our results highlight the need for the provision of sufficient European Union funding to appropriately continue to implement an effective agri-environment scheme AES [55]. Moreover, the carrying capacity of this area could be maintained or even increased by the continuous maintenance and provisioning of artificial nest-sites. In the Vale do Guadiana SPA (where the Mértola colony is located) land use changes are driven by the decisions of farmers and landowners and by the availability of different policy initiatives. Without the existence of an AES or the interdiction of afforestation the entire area has been suffering a continuous habitat loss and fragmentation. Currently, the provisioning of artificial nests in the Mértola colony will not contribute to reverse the declining population trend. Raising the carrying capacity of an area for breeding birds through an increase in food supply is more difficult than managing nest-sites as it usually entails changing the land use so as to promote an increase in prey. Often, the best option for fragmented or intensified areas is to find the remaining existing areas of good habitat and prevent their further degradation [14]. In the Vale do Guadiana SPA two remaining areas (with ca 1500 ha each) of high quality habitat but with no lesser kestrels or habitat management were identified [65]. The shortage of nest-sites was rectified by adding sites artificially in 2006 and immediately the area was colonized by breeding lesser kestrels. In these remaining areas, the implementation of an AES similar to the existing in Castro Verde SPA and importantly the interdiction of afforestation could prevent the direction of vegetation succession and enhance habitat availability and prey abundance for lesser kestrels.

The Birds directive does not have any tied funding allocated to it and SPAs are greatly dependent on European funds such as the European Agricultural Fund for Rural Development (EAFRD) or LIFE Programmes to support environmental and nature conservation. Increasing the conservation cost-effectiveness of these funding instruments, e.g. by avoiding the application of contradictory land use policies (e.g. [22]) and by correctly identifying key resources of target populations is a major challenge to both researchers and decision makers.
